# Novel twin-roll-cast Ti/Al clad sheets with excellent tensile properties

**DOI:** 10.1038/s41598-017-08681-9

**Published:** 2017-08-14

**Authors:** Dae Woong Kim, Dong Ho Lee, Jung-Su Kim, Seok Su Sohn, Hyoung Seop Kim, Sunghak Lee

**Affiliations:** Center for Advanced Aerospace Materials, Pohang University of Science and Technology, Pohang, 790-784 Korea

## Abstract

Pure Ti or Ti alloys are recently spot-lighted in construction industries because they have excellent resistance to corrosions, chemicals, and climates as well as various coloring characteristics, but their wide applications are postponed by their expensiveness and poor formability. We present a new fabrication process of Ti/Al clad sheets by bonding a thin Ti sheet on to a 5052 Al alloy melt during vertical-twin-roll casting. This process has merits of reduced production costs as well as improved tensile properties. In the as-twin-roll-cast clad sheet, the homogeneously cast microstructure existed in the Al alloy substrate side, while the Ti/Al interface did not contain any reaction products, pores, cracks, or lateral delamination, which indicated the successful twin-roll casting. When this sheet was annealed at 350 °C~600 °C, the metallurgical bonding was expanded by interfacial diffusion, thereby leading to improvement in tensile properties over those calculated by a rule of mixtures. The ductility was also improved over that of 5052-O Al alloy (25%) or pure Ti (25%) by synergic effect of homogeneous deformation due to excellent Ti/Al bonding. This work provides new applications of Ti/Al clad sheets to lightweight-alloy clad sheets requiring excellent formability and corrosion resistance as well as alloy cost saving.

## Introduction

Pure Ti or Ti alloys have many attractive properties such as high specific strength and stiffness due to relatively low density and excellent resistance to corrosion and oxidation due to oxide-film formation^1–8^. Recently, they are spot-lighted in construction industries because they have excellent resistance to corrosions, chemicals, and climates as well as various coloring characteristics obtained from surface treatments. However, wide applications to these industries are postponed by their expensiveness and poor room-temperature formability. Considering that such advantages mostly stand out in surface regions, efforts to address shortcomings and to widen applications have been actively made by fabricating thin Ti sheets clad with other inexpensive and readily-deformable stainless steel or Al alloy sheets *via* solid/solid-state bonding methods^9–15^. Since these methods are usually proceeded under high temperatures and reduction ratios, the interfacial bonding is often deteriorated by forming brittle interfacial layers composed of intermetallic compounds^16–20^. Their production costs are also considerably high because commercial sheet products of Ti, Al, or stainless steel should be used.

As a promising approach, instead of the solid/solid-state bonding, a liquid/solid-state bonding method, *i.e*., cladding by twin-roll casting which is a method for continuously casting thin strips or sheets, has been suggested for Ti/Al clad sheets in the present study^21, 22^. The solidification rate is higher in the twin-roll casting than in the conventional continuous casting, and thus can be utilized as an environmentally conscious process because of refined cast microstructures and reduced micro-segregations^23^. If the cladding of thin Ti sheet on to Al melt is achieved by the twin-roll casting, shortcomings of pure Ti or Ti alloys for applications to construction materials or components are effectively solved, while production costs are reduced. However, very few researches on liquid/sold bonding of Ti sheet and Al melt during the twin-roll casting have been performed^24^. Since the casting proceeds under a direct contact of a roll with a solidified Al melt shell and a thin Ti sheet, different approaches on the liquid/solid bonding are needed. Even after the twin-roll casting would be successfully made, the bonding of Ti/Al clad sheets should be further enhanced because it is insufficient, but effects of microstructural and process parameters on the liquid/solid bonding have not been sufficiently studied yet^24^. Here we present a new fabrication process of Ti/Al clad sheets by vertical twin-roll casting and annealing, and analyze their microstructures and tensile properties. This process has merits of reduced production costs as well as improved tensile properties. Appropriate microstructural and process parameters of Ti/Al clad sheets are also suggested.

## Results

### Microstructures of twin-roll-cast Ti/Al clad sheets

**Figure 1:**
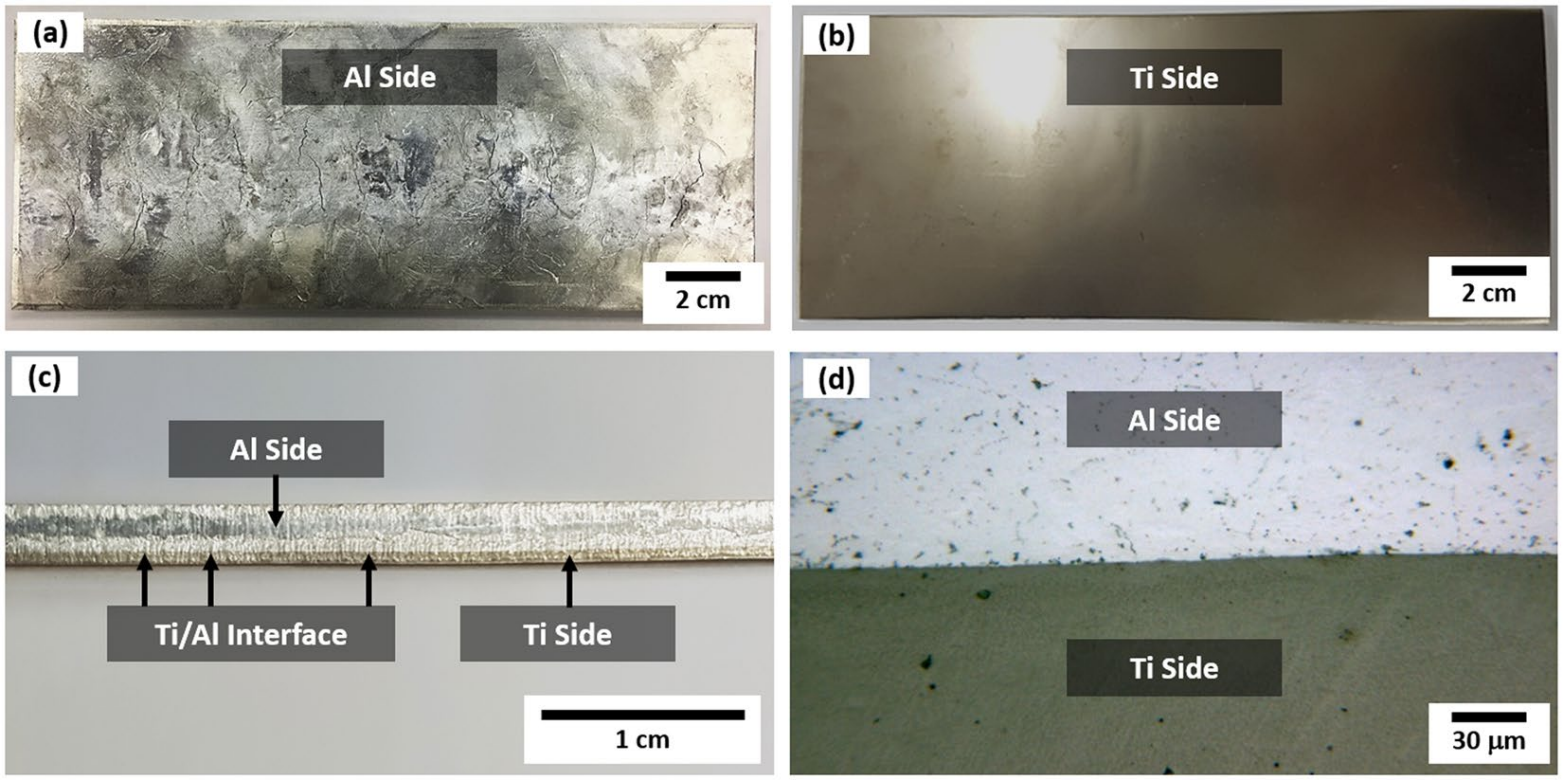
Overall shape of as-twin-roll-cast Ti/Al clad sheet. Low-magnification optical photographs of (**a**) 5052 Al alloy substrate side, (**b**) Ti side, and (**c**) and (**d**) Ti/Al interface of the as-twin-roll-cast Ti/Al clad specimen made by optimum parameters (induction-melting temperature of Al alloy; 680 °C, twin-roll speed; 30 mm/sec, twin-roll gap; 3.0 mm, thickness of pure Ti sheet; 0.5 mm).

Low-magnification optical photographs of the as-cast Ti/Al clad specimen made by optimum parameters (induction-melting temperature of Al alloy; 680 °C, twin-roll speed; 30 mm/sec, twin-roll gap; 3.0 mm, thickness of pure Ti sheet; 0.5 mm) are shown in Fig. 1(a) through (d). The as-cast microstructure in the 5052 Al substrate side is relatively homogeneous (Fig. 1(a)), while wrinkles are not formed in the Ti side (Fig. 1(b)). A Ti/Al interface is observed in a side view of the clad specimen (Fig. 1(c)). When the side view is magnified, cracks, pores, interfacial delamination, or reaction products are not found (Fig. 1(d)), which indicates the successful vertical-twin-roll casting of Ti/Al clad specimens. The thickness of the Ti layer (0.5 mm) does not decrease because the Ti sheet was not molten or reduced during the twin-roll casting.

**Figure 2:**
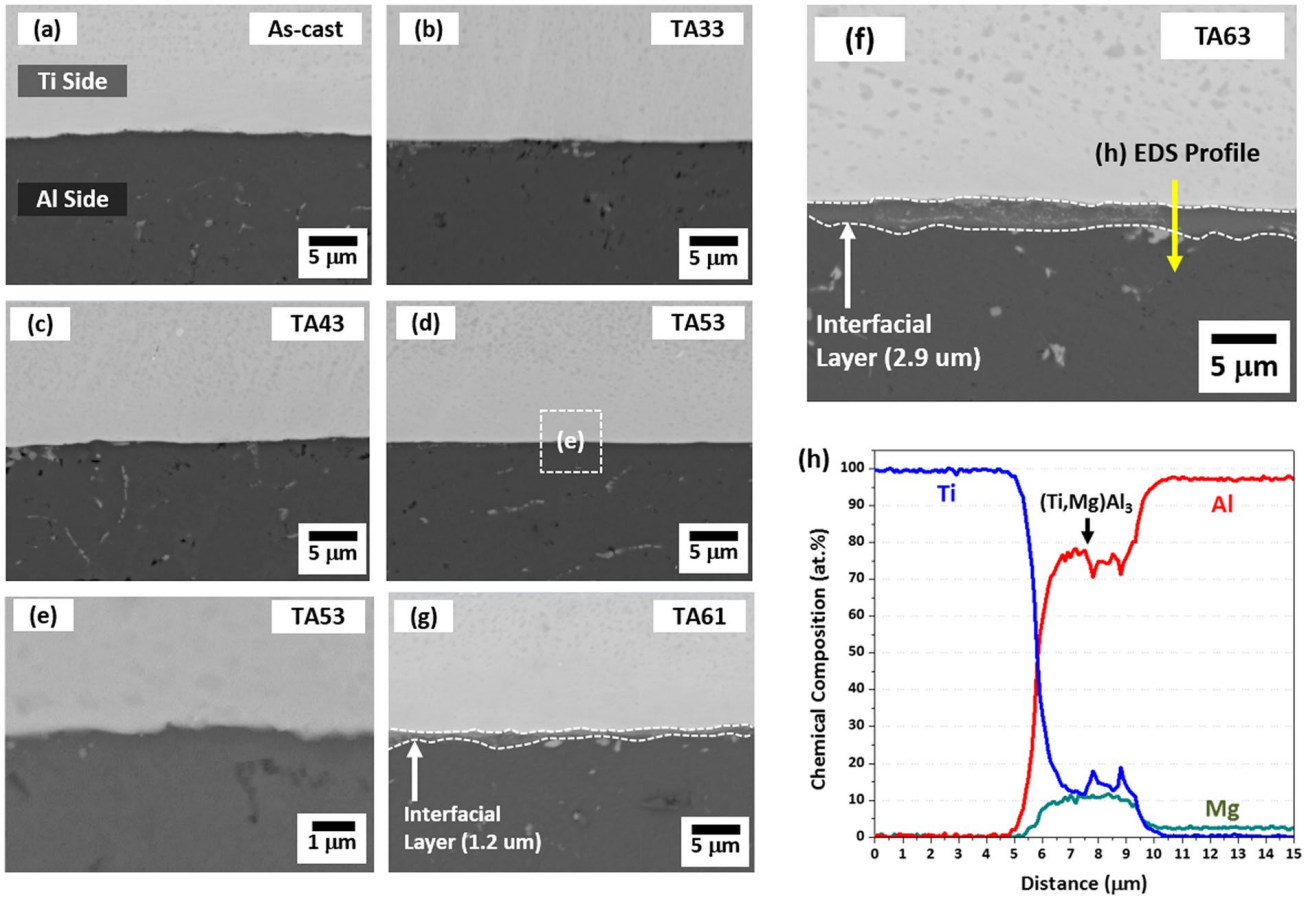
Microstructures of twin-roll-cast and annealed Ti/Al clad sheets. SEM micrographs of the cross-sectional area of the (**a**) as-cast specimen and (**b–f**) specimens annealed at 350 °C, 400 °C, 500 °C, and 600 °C for 30 min (TA33, TA43, TA53, and TA63 specimens, respectively). The interfacial reaction layer thickness is about 2.9 μm, which is thicker than that (1.2 μm) of the TA61 specimen, as shown in (**g**). The EDS data of the interfacial reaction layer of the TA63 specimen is shown in (**h**), and indicate that the layer is composed of (Ti,Mg)Al_3_.

**Figure 3:**
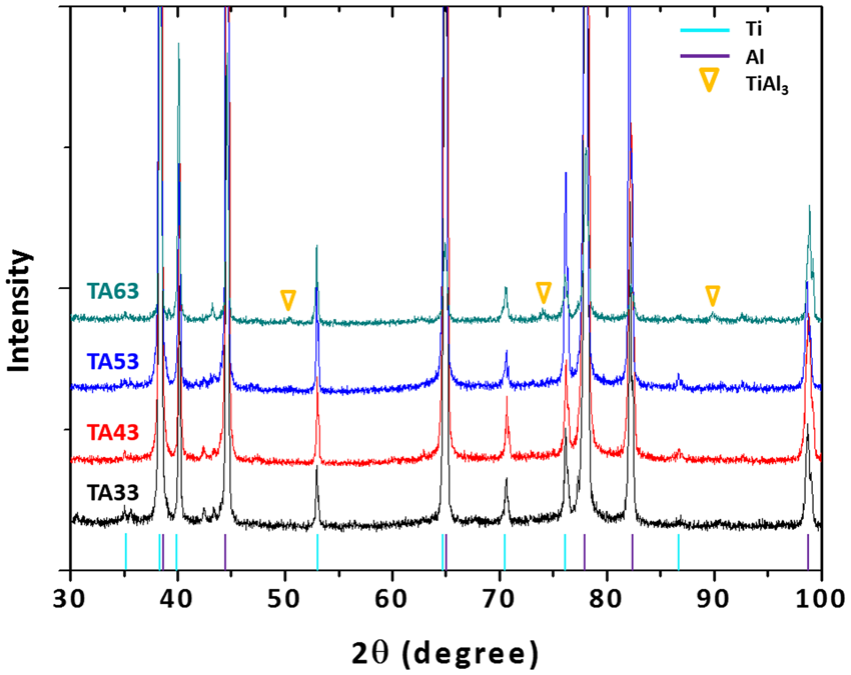
Existence of interfacial reaction layer (TiAl_3_) in 600 °C-annealed specimen. X-ray diffraction peaks of the TA33, TA43, TA53, and TA63 specimens. In the TA63 specimen, peaks of a reaction product, *i.e*., TiAl_3_, are formed, whereas they are not found in TA33, TA43, and TA53 specimens.

Figure 2(a) through (e) show SEM micrographs of the cross-sectional area of the as-cast specimen and specimens annealed at 350 °C, 400 °C, 500 °C, and 600 °C for 30 min (TA33, TA43, TA53, and TA63 specimens, respectively). In the as-cast specimen, the Al cast microstructure is relatively homogeneous, and reaction products are not found along the Ti/Al interface (Fig. 2(a)). The basic microstructures of the 350 °C-, 400 °C-, and 500 °C-annealed specimens (TA33, TA43, and TA53 specimens, respectively) is similar to that of the as-cast specimen, although residual stresses might be relieved (Fig. 2(b) through (d)). Figure 2(e) shows a higher-magnification SEM micrograph of the TA53 specimen. There are no reaction products in this TA53 specimen, which implies that the 500 °C-annealing is not enough to form reaction products. In the TA63 specimen, however, a reaction product is formed along the Ti/Al interface (Fig. 2(f)), whereas it is not found in TA33, TA43, and TA53 specimens (Fig. 2(b) through (e)). The thickness of this interfacial reaction layer is about 2.9 μm, which is thicker than that (1.2 μm) of the TA61 specimen (specimen annealed at 600 °C for 10 min, Fig. 2(g)). When this interfacial layer is quantitatively analyzed along a yellow arrow in Fig. 2(f) by the EDS, the average contents of Ti, Al, and Mg are 14.0%, 75.4%, and 10.6% (atomic percent), respectively (Fig. 2(h)). This EDS data indicates that the layer is composed of (Ti,Mg)Al_3_ intermetallic compound in which some Ti content of TiAl_3_ (a typical intermetallic compound in Ti-Al phase diagram^25^) is replaced by Mg according to the interfacial diffusion of Mg existed in the 5052 Al alloy. The existence of TiAl_3_ is also confirmed by the XRD data, as shown in Fig. 3. This TiAl_3_ is readily formed at relatively low temperatures among various Ti-Al intermetallic compounds such as TiAl_3_, TiAl, and Ti_3_Al^25^.

### Room-temperature tensile properties

Figure 3(a) through (d) show room-temperature engineering tensile stress-strain curves of the as-cast and annealed specimens, and the yield strength, ultimate tensile strength, and elongation are summarized in Table 1. The as-cast specimen is not much strained, thereby showing very low strength and elongation (Fig. 3(a)). The TA33 specimen shows much higher strength and elongation than the as-cast specimen, but the stress decreases rather rapidly after the peak stress at the strain of 15%. The yield strength, tensile strength, and elongation of the TA36 specimen are 120 MPa, 217 MPa, and 54.5%, respectively, which are improved from those of the TA33 specimen. Here, rapid stress drops are found in the stress-strain curves as marked by brown and green arrows. The TA41 and TA43 specimens show similar tensile properties to those of the TA36 specimens, together with some stress drops (Fig. 3(b)). In the TA46 specimen, the stress decreases rapidly after the stress reaches the ultimate tensile stress. In the 500 °C-annealing specimens, the yield and tensile strengths are similar to those of the 400 °C-annealing specimens, and the elongation increases with increasing annealing time (Fig. 3(c)). Overall tensile properties and stress-strain curve shapes of the TA61 specimen are similar to those of the TA36 or TA43 specimen, but the elongation is seriously reduced to 17% in the TA63 specimen (Fig. 3(d)). The TA31and TA66 specimens are not tensile-tested because it is easily expected that tensile properties of the TA31 specimen lie between those of the as-cast and TA33 specimens, while those of the TA66 specimen is lower than those of the TA63 specimen.

**Table 1:** Room-temperature tensile properties of the twin-roll-cast Ti/Al clad specimens.

Clad Specimen	Yield Strength (MPa)	Tensile Strength (MPa)	Total Elongation (%)	Strain at Cracking (%)	Strain at Interfacial Delamination (%)
As Cast	99 ± 5	152 ± 7	17.0 ± 1.2	**7.8 ± 1.3** ^*^	12.5 ± 0.8
TA33	116 ± 7	212 ± 5	36.0 ± 2.9	**21.6 ± 4.4** ^*^	31.3 ± 1.0
TA36	120 ± 4	217 ± 8	54.5 ± 2.2	**43.4 ± 3.4** ^*^	51.4 ± 3.3
TA41	118 ± 7	211 ± 7	41.1 ± 0.8	—	**30.6 ± 0.8** ^*^
TA43	132 ± 5	218 ± 9	46.1 ± 1.8	—	**40.5 ± 1.5** ^*^
TA46	123 ± 4	213 ± 6	44.3 ± 2.5	**24.5 ± 2.2** ^*^	27.6 ± 1.7
TA51	115 ± 3	227 ± 2	35.0 ± 1.3	**27.4 ± 2.9** ^*^	32.0 ± 1.2
TA53	118 ± 2	229 ± 4	44.5 ± 1.7	—	**29.2 ± 3.3** ^*^
TA56	115 ± 3	221 ± 2	50.7 ± 2.2	—	**30.7 ± 2.0** ^*^
TA61	91 ± 3	214 ± 5	48.8 ± 1.1	—	**27.9 ± 1.1** ^*^
TA63	83 ± 3	198 ± 3	17.4 ± 2.1	—	**17.2 ± 0.5** ^*^
H32-treated	151 ± 4	234 ± 6	38.4 ± 3.3	—	**28.8 ± 2.8** ^*^

*Effective ductility.

### Digital images of strain distribution taken during tensile tests

**Figure 4:**
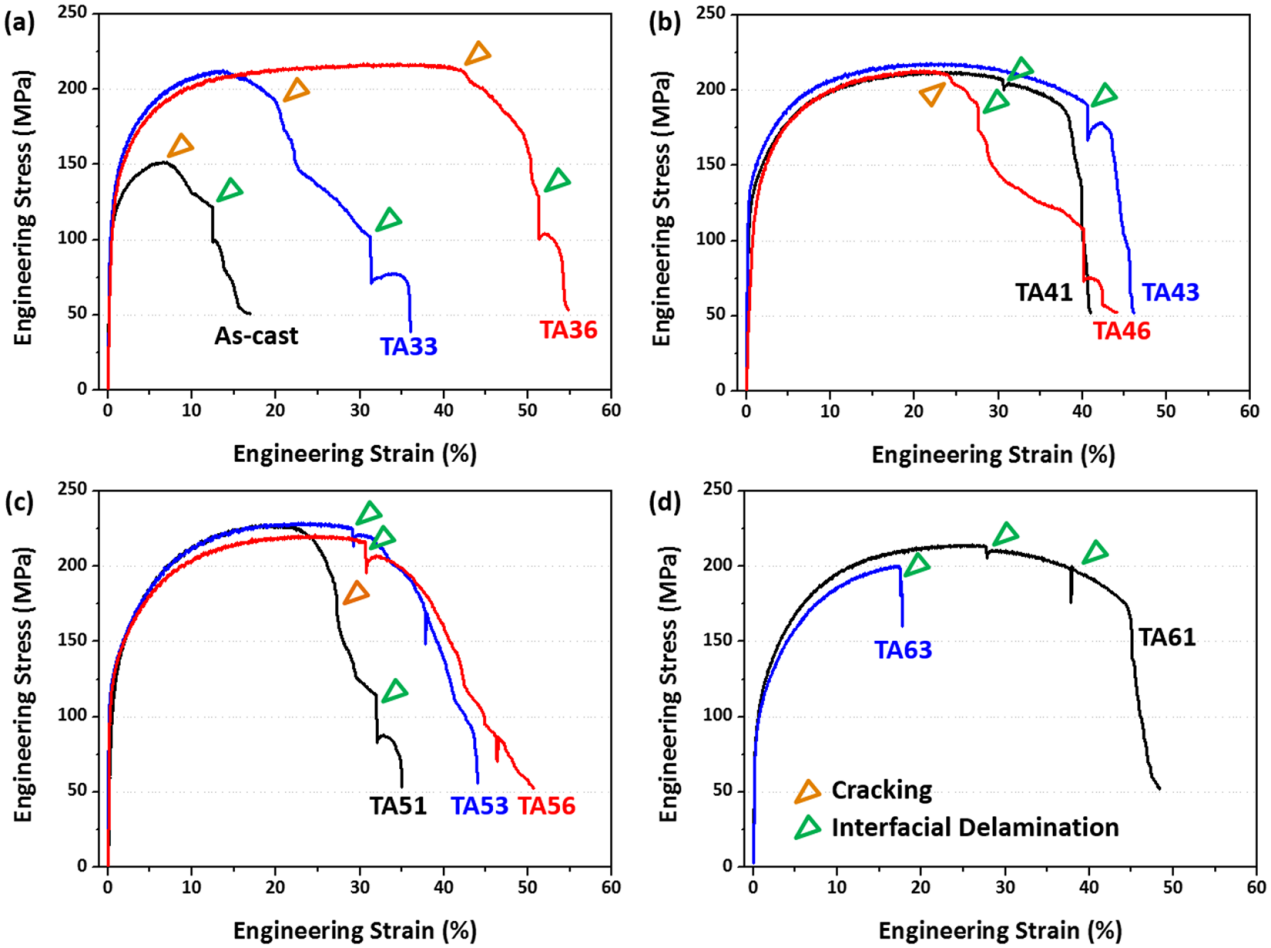
Room-temperature engineering stress-strain curves. Tensile stress-strain curves of the (**a**) as-cast and 350 °C-annealed, (**b**) 400 °C-annealed, (**c**) 500 °C-annealed, and (**d**) 600 °C-annealed specimens. Strains at the cracking point and interfacial delamination point are indicated by brown and green arrows, respectively.

**Figure 5:**
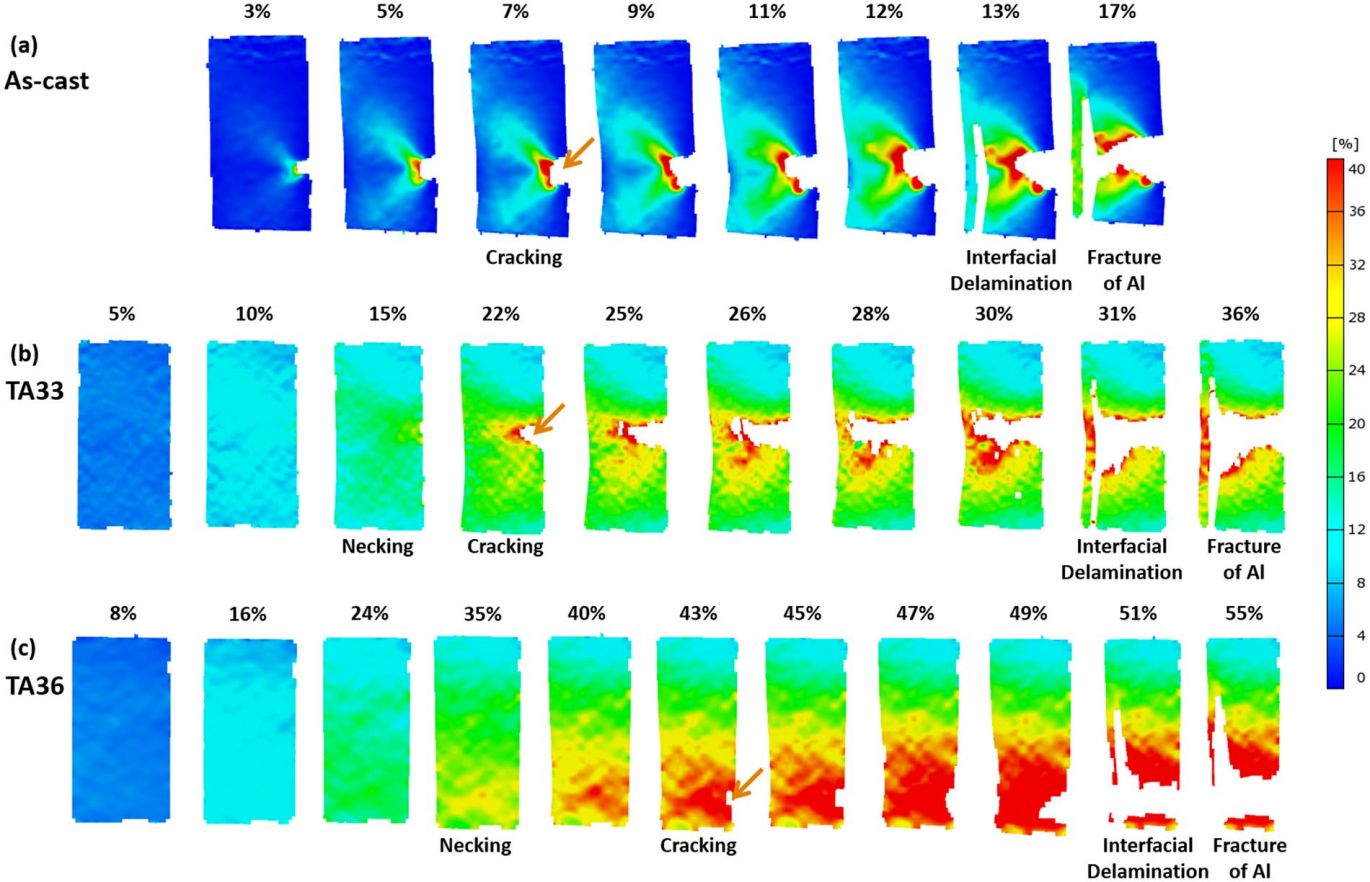
Digital images of strain distribution taken during tensile tests. Digital images of the (**a**) as-cast, (**b**) TA33, (**c**) TA36 specimens. Strains at the necking, cracking, and interfacial delamination points are described above each digital image.

Since rapid stress drops are frequently observed in the stress-strain curves (Fig. 4(a) through (d)), the DIC technique was utilized simultaneously with tensile tests. Figure 5 through 8 show digital images of strain distribution taken during tensile tests of the as-cast and annealed specimens. These digital image data were selected from several hundreds of high-resolution images. In the as-cast specimen, eight images are shown in the strain range from 3% to fracture strain (17%) because the specimen is abruptly cracked in the Al side right after the tensile deformation (Fig. 5(a)). The Ti/Al interface is delaminated at the strain of 13%, thereby leading to the abrupt stress drop (Fig. 4(a)). When the specimen is tensile-deformed further, the crack connects with the delaminated interface to reach the failure. In the TA33 specimen, ten images are selected from the strain of 5% to the fracture strain (36%) (Fig. 5(b)). The necking starts at the strain of 15%, after which the cracking occurs at the localized central area of the Al side, as indicated by a brown arrow in Fig. 5(b). This cracking occurring at the strain of 22% leads to a stress drop in the stress-strain curve (brown arrow mark in Fig. 4(a)), and the crack keeps blunting and growing into the interior with increasing strain to 30%. At the strain of 31%, the Ti/Al interface is delaminated when the crack is connected with the interface, thereby leading to a rapid stress drop in the stress-strain curve (green arrow mark in Fig. 4(a)). When the TA33 specimen is strained further to 36%, the specimen is fractured. Figure 5(c) shows the tensile deformation behavior of the TA36 specimen, which is almost similar to that of the TA33 specimen. The necking, cracking, and interfacial delamination occur at the strains of 35%, 43%, and 51%, respectively, which are higher than those of the TA33 specimen.

**Figure 6:**
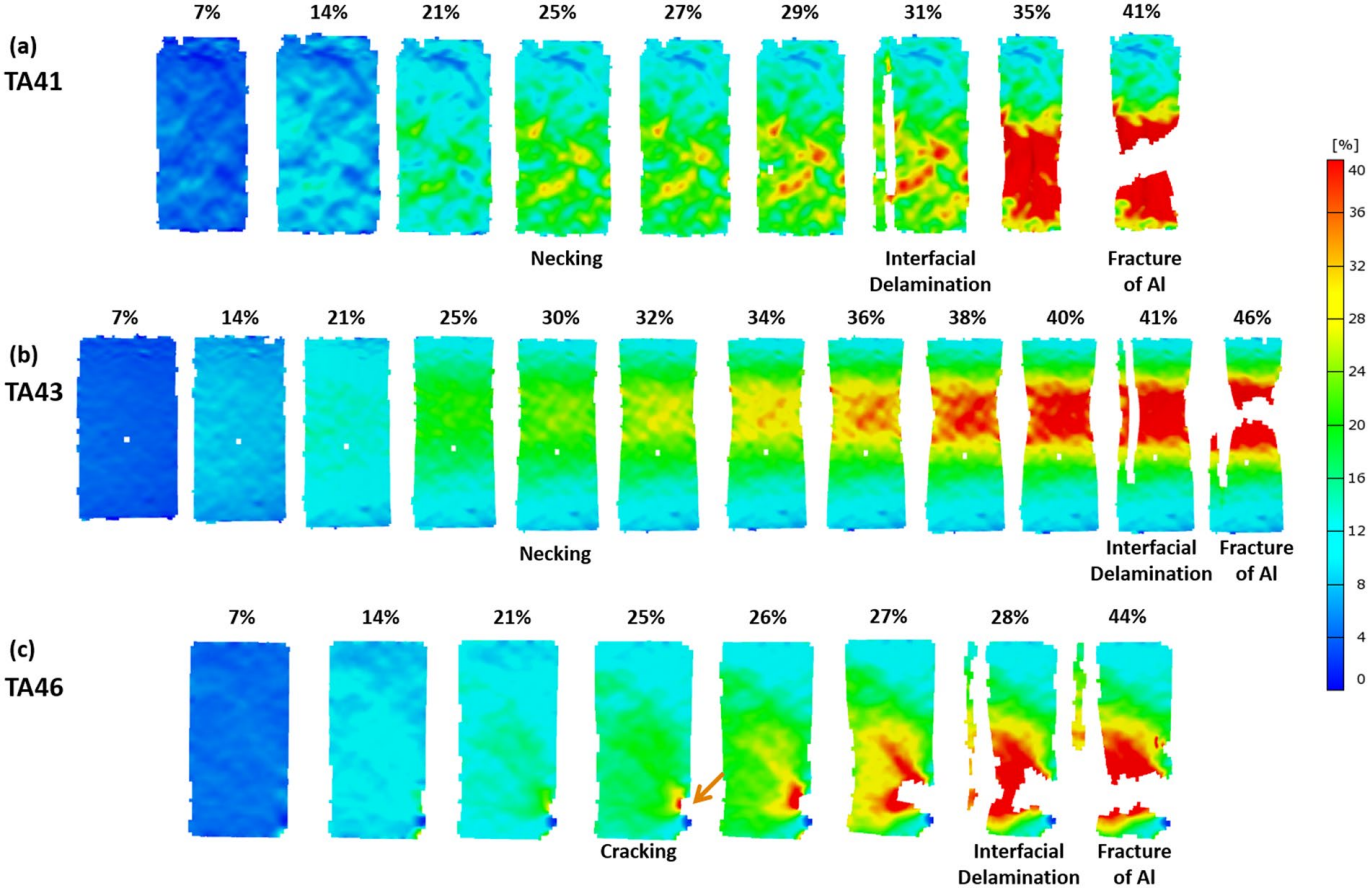
Digital images of strain distribution taken during tensile tests. Digital images of the (**a**) TA41, (**b**) T43, (**c**) TA46 specimens. Strains at the necking, cracking, and interfacial delamination points are described above each digital image.

The TA41 specimen is homogeneously deformed in the strain range of 7~25%, and the necking starts at the strain of 25% (Fig. 6(a)). After the necking, the tensile deformation is concentrated to reach the maximum local strain of about 29%. At the strain of 31%, the interfacial delamination occurs, which is matched with a stress drop, as indicated by a green arrow in Fig. 4(b). After the delamination, the Al substrate is tensioned further to reach the fracture strain (41%). The TA43 specimen tends to be more homogeneously deformed than the TA41 specimen as the necking, interfacial delamination, and fracture occur at the strains of 30%, 41%, and 46%, respectively (Fig. 6(b)). The TA46 specimen shows a similar deformation behavior to the TA43 specimen because the cracking occurs at the strain of 25% (Fig. 6(c)), which is much lower than that of the TA36 specimen (43%).

**Figure 7:**
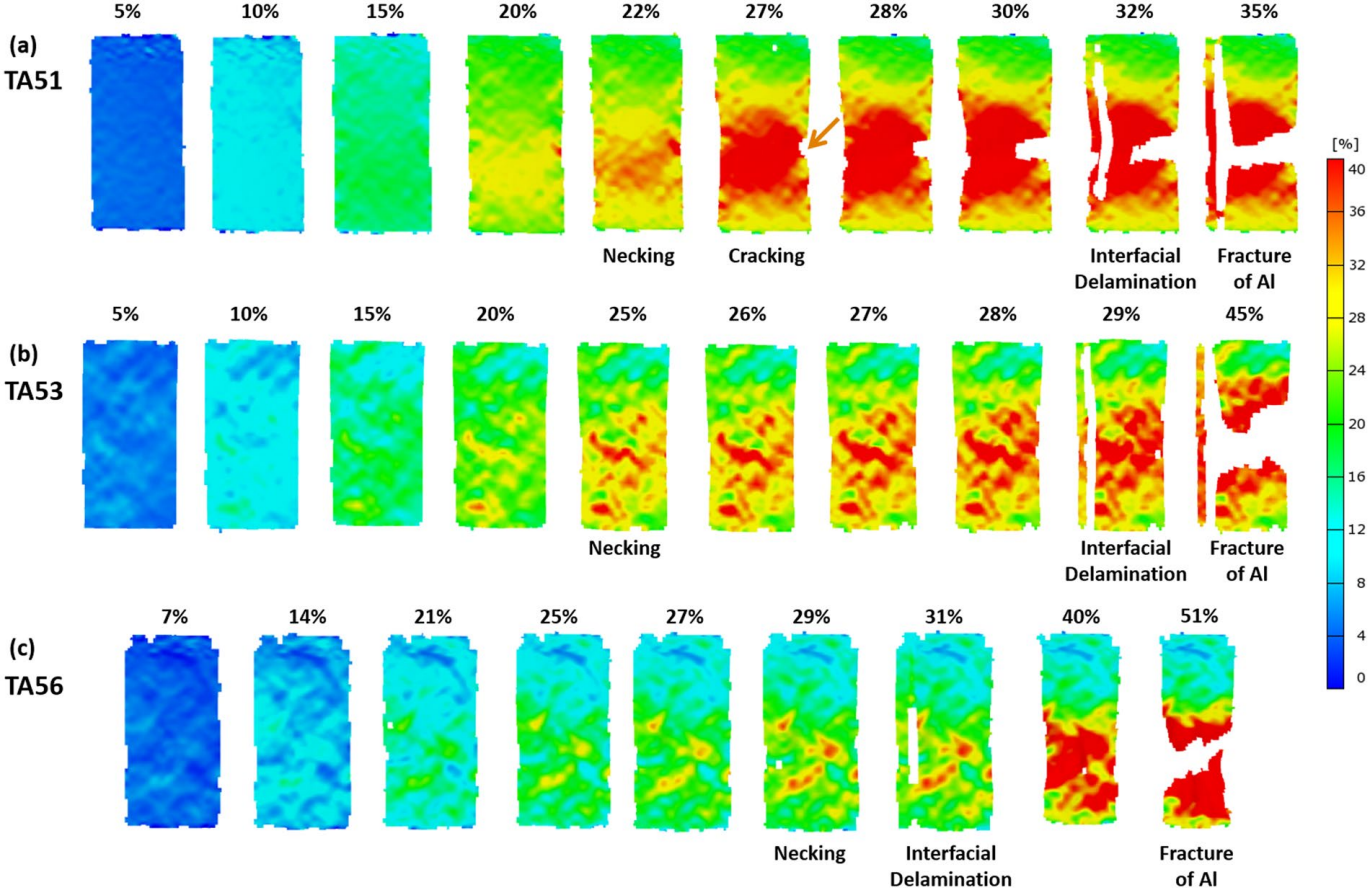
Digital images of strain distribution taken during tensile tests. Digital images of the (**a**) TA51, (**b**) TA53, (**c**) TA56 specimens. Strains at the necking, cracking, and interfacial delamination points are described above each digital image.

In the TA51 specimen, the necking starts at the strain of 22%, and then the cracking, interfacial delamination, and fracture follow at the strain of 27%, 32, and 35%, respectively (Fig. 7(a)). In the TA53 specimen, the deformation is concentrated in a shear mode in a relatively wide area after the necking (Fig. 7(b)), which is somewhat different from the deformation mode of the other specimens. The interfacial delamination firstly occurs at the strain of 29%, which is lower than that of the TA43 specimen. The tensile deformation behavior of the TA56 specimen is almost similar to that of the TA53 specimen, although the shear-mode strain concentration is not obvious (Fig. 7(c)).

**Figure 8:**
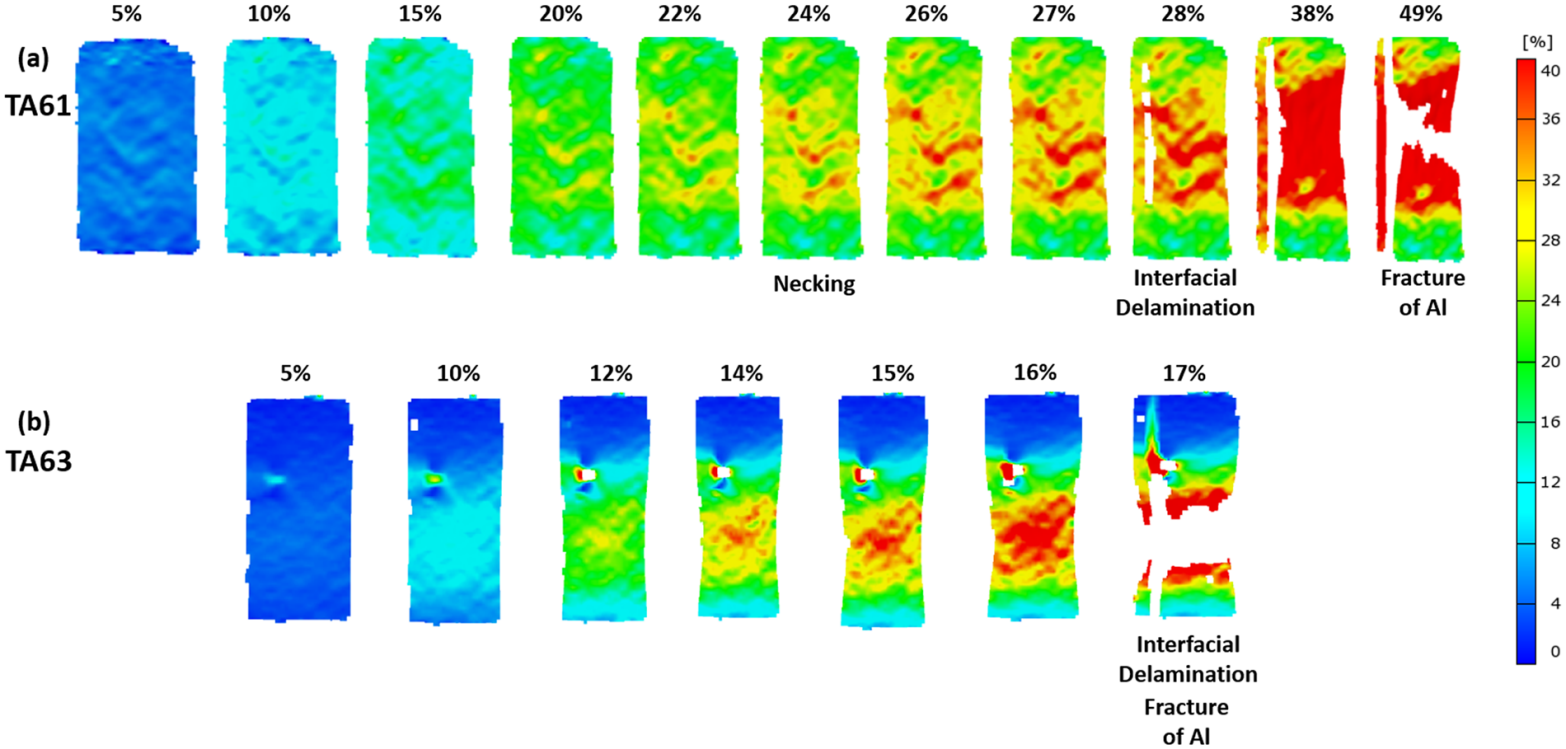
Digital images of strain distribution taken during tensile tests. Digital images of the (**a**) TA61, (**b**) TA63 specimens. Strains at the necking, cracking, and interfacial delamination points are described above each digital image.

The TA61 specimen shows the almost similar tensile deformation behavior to the TA53 specimen (Fig. 8(a)). The necking and interfacial delamination occur at the strains of 24% and 28%, respectively, which are lower than those of the TA43 or TA53 specimen. After the first interfacial delamination, the A61 specimen is deformed further to the strain of 38%, at which the second rapid stress drop occurs (Fig. 3(d)), and then reaches the fracture strain of 49%. In the TA63 specimen, the interfacial delamination starts at the low strain of 12%, which results in the early fracture at the strain of 17% (Fig. 8(b)).

## Discussion

### Effective ductility in tensile stress-strain curves

The cast Al microstructure of the as-cast Ti/Al clad specimen is relatively homogeneous without forming any interfacial reaction products (Fig. 2(a)). No interfacial reaction products might be attributed to the insufficient diffusion bonding during the vertical-twin-roll-casting process, thereby leading to poor strengths and ductility (Fig. 3(a) and Table 1). Thus, the as-cast specimen must be annealed to enhance the interfacial bonding and to relieve stresses formed in the Al side^26^. After the annealing at 350 °C~500 °C, the interfacial diffusion is promoted to improve the metallurgical bonding and consequent tensile properties, although reaction products are not clearly visible in the Ti/Al interface (Fig. 2(b) through (d)).

Tensile properties of the annealed specimens are dependent upon necking, cracking, and delamination phenomena appearing during tensile tests (Fig. 4(a) through (e)) as well as annealing temperature and time. These phenomena directly affect the stress-strain behavior. According to the DIC results of the tensioned TA33 specimen (Fig. 4(a)), the homogeneous deformation occurs while the strain increases continuously to 15%, and the necking, cracking, and interfacial delamination appear sequentially at the strains of 15%, 22%, and 31%, respectively. Though this specimen is deformed further until the fracture strain of 35%, the additional ductility after the cracking has no meaning in the tensile property evaluation because the clad specimen cracked in the Al side is not useful any more. In the TA43 and TA53 specimens, the cracking does not occur, but the interfacial delamination occurs at the strains of 41% and 29%, respectively, after which the TA43 and TA53 specimens are also useless. Thus, the strain at the cracking point or interfacial delamination point is more meaningful or effective in the property evaluation of the Ti/Al clad specimens than the fracture strain or total elongation, and these strain data are added in Table 1. The effective ductility can be the strain at the cracking point or interfacial delamination point because the cracking occurs earlier than the interfacial delamination. For example, the effective ductility is the strain at the cracking point in the TA36 specimen where both cracking and delamination appear, while it becomes the strain at the interfacial delamination point in the TA43 specimen where the delamination appears without the cracking. These effective ductility data are denoted by star-marks in Table 1.

The effective ductility, *i.e*., the strain at the cracking point or interfacial delamination point, is highest in the TA36 specimen, and decreases in the order of TA43, TA56, TA41, TA53, TA51, TA46, TA33, and as-cast specimens (Table 1). When considering both strength and ductility together, overall tensile properties of the TA43 specimen might be similar to those of the TA36 specimen because the yield strength of the TA43 specimen is higher by 12 MPa than that of the TA36 specimen. The 500 °C-annealed specimens show the lower yield strength (115~118 MPa), higher tensile strength (221~229 MPa), and lower ductility (28~31%) than the TA36 or TA43 specimen. Their overall tensile properties are somewhat lower than those of the TA36 or TA43 specimen.

### Effects of interfacial (Ti,Mg)Al_3_ intermetallic compound formed in 600 °C-annealed specimens on tensile properties

The present twin-roll-cast clad sheets were annealed at 350~600 °C for 10~60 min. In the annealing temperature range, the phase transformation does not occur in the pure Ti and 5052 Al alloy layers^25^. However, an interfacial reaction phase of (Ti,Mg)Al_3_ is formed at the Ti/Al interface of the 600 °C-annealed specimens, as shown in Fig. 2(e) and (f), whereas it is not found in the 350~500 °C-annealed specimens. It is known that interfacial intermetallic compounds generally help to improve the interfacial bonding, but that they often deteriorate tensile or interfacial bonding properties because they have inherently brittle characteristics^18^. When their layers are thicker than a certain thickness level, tensile properties as well as interfacial bonding can be enhanced^27^. In the TA61 specimen, tensile properties are slightly better than those of the TA43 specimen if the interfacial delamination is not considered (Table 1). This is because the (Ti,Mg)Al_3_ layer formed along the Ti/Al interface (Fig. 2(e)) can improve tensile properties when its thickness is optimally controlled. In the TA63 specimen, the interfacial (Ti,Mg)Al_3_ layer is grown to 2.9 μm, and deteriorates overall tensile properties because it is too thickened. However, it is relatively easily cracked to induce an early interfacial delamination at the strains of 28% and 17% in the TA61 and TA63 specimens, respectively, during the tensile test (Fig. 3(d)). According to the importance of the effective ductility (interfacial delamination strain), the TA61 and TA63 specimens have the lower tensile properties than the TA36 or TA43 specimen. In fact, the objective of the 600 °C-annealing is not to improve tensile properties, but to examine effects of formation of Ti-Al intermetallic compounds on tensile properties because an O-temper treatment of commercial 5052 Al alloy (annealing at 345~415 °C) is considered practically in this study^28^.

### Comparison of measured and calculated tensile properties by a rule of mixtures

The engineering yield and tensile strengths of the Ti/Al clad specimens are calculated by a rule of mixtures based on volume fraction without considering the interfacial interaction between Ti and Al elements. Thus, this strength calculation is not a theoretically established calculation approach but a simple approach for the property comparison purpose. For the calculation, conventional tensile properties of a 5052-O Al alloy (thickness; 2.5 mm, yield strength; 90 MPa, tensile strength; 195 MPa)^29^ and a pure Ti (675 °C-annealed, thickness; 0.5 mm, yield strength; 170 MPa, tensile strength; 240 MPa)^30^ are used. The yield and tensile strengths of the TA36 and TA43 specimens (120 MPa and 217 MPa, and 132 MPa and 218 MPa, respectively (Table 1)) are higher than the calculated yield and tensile strengths (103.3 MPa and 202.5 MPa, respectively). In addition, the effective ductility of the TA36 and TA43 specimens (43.4% and 40.5%, respectively) is much higher than that of 5052-O Al alloy (25%)^28^ or pure Ti (25%)^31^. This is attributed to a synergic effect of the delay of tensile necking due to homogeneous deformation (Fig. 4(b) and (c)) when the 5052 Al and pure Ti are well bonded. The enhanced ductility is also closely related with to the presence of well-bonded Ti layer in terms of mechanisms such as homogeneous sharing of applied loads and deformation in both Al and Ti sides.

**Figure 9:**
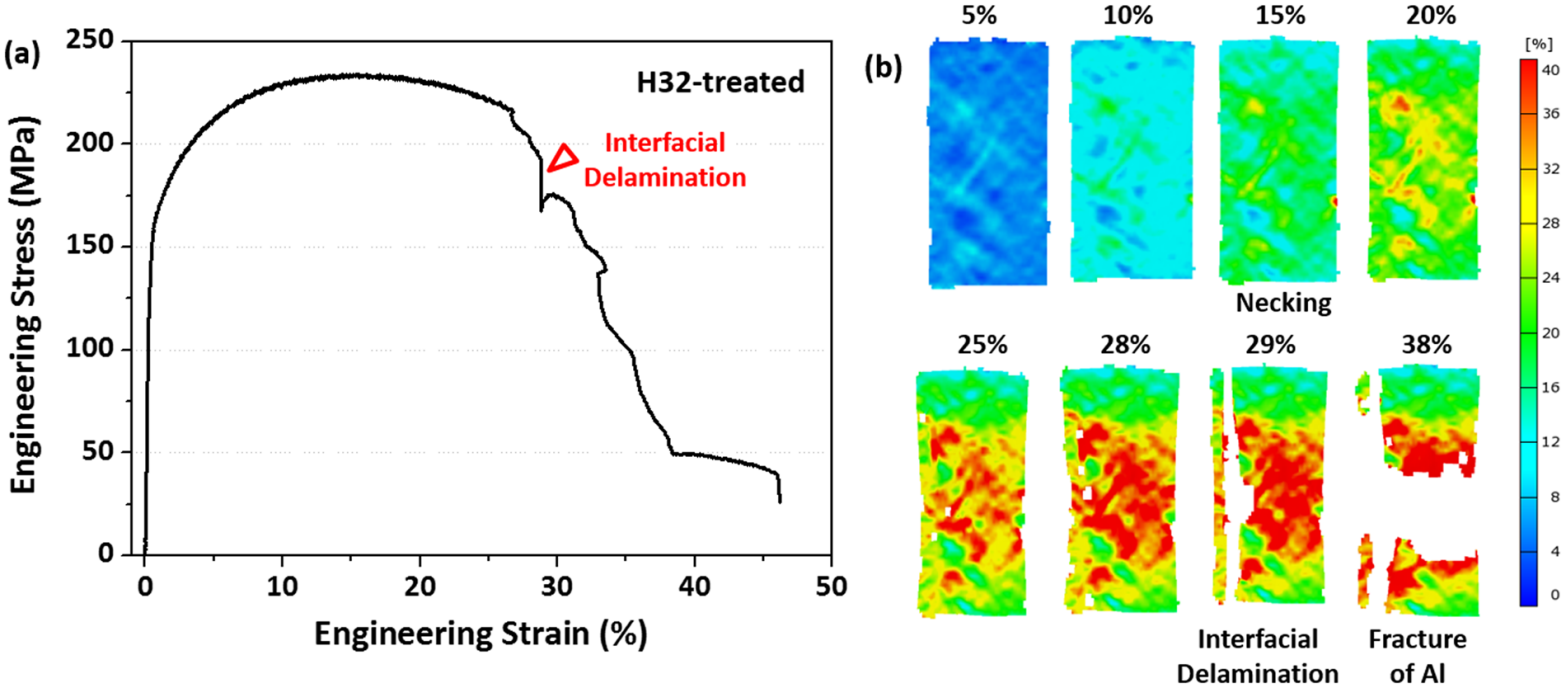
Room-temperature engineering stress-strain curve and digital images of strain distribution of the H32-treated Ti/Al clad sheet. Tensile stress-strain curve and digital image of the Ti/Al clad specimen subjected to H32 treatment of 5052 Al alloy (18.8%-cold-rolling and 400 °C-30-min-annealing). The yield strength, tensile strength, total elongation, and effective elongation (strain at the interfacial delamination point) of the H32-treated clad specimen are 151 MPa, 234 MPa, 38%, and 29%, respectively.

Since a cold-rolled and stress-relief annealing condition of 5052 Al alloy, *e.g*., H32 treatment (18.8%-cold-rolling and (345~415 °C)-annealing), is often used for the high-strength applications, the as-cast Ti/Al clad specimen was 18.8%-cold-rolled and 400 °C-30-min-annealed. The stress-strain curve, DIC data, and measured tensile properties are shown in Fig. 9(a) and (b) and Table 1. The yield strength, tensile strength, total elongation, and effective elongation of the H32-treated clad specimen are 151 MPa, 234 MPa, 38.4%, and 28.8%, respectively. The yield and tensile strengths are comparable to those (190.8 MPa and 231.7 MPa, respectively) calculated from tensile properties of a 5052-H32 Al alloy (thickness; 2.5 mm, 151 MPa and 234 MPa, respectively) and a pure Ti (675 °C-annealed, thickness; 0.5 mm, 170 MPa and 234 MPa, respectively)^30^. The effective ductility (28.8%) is also higher than that of the 5052-H32 Al (12%)^29^ or pure Ti (25%)^26^. Thus, this H32-treated clad specimen can be readily used for applications requiring higher yield and tensile strengths together with excellent ductility.

Based on findings of this study on Ti/Al clad sheets having excellent tensile properties, the fabrication process composed of vertical-twin-roll casting and stress-relief annealing as well as the interfacial bonding behavior are well understood. The as-twin-roll-cast Ti/Al clad sheet has a weak interface because of insufficient diffusion bonding, but its interfacial bonding and tensile properties are dramatically improved after the stress-relief annealing. Their tensile properties are higher than those calculated by a rule of mixtures or those of individual 5052 Al or pure Ti, which shows a synergic effect of homogeneous deformation due to excellent Ti/Al bonding. In particular, the improved ductility can be plausibly explained by the well-bonded Ti layer because it uniformly accommodates applied loads and homogeneously deforms with the Al layer which is verified by macroscopic local strain distribution analyses, *i.e*., DIC technique. In fact, the tensile deformation and interfacial debonding behavior occurring during tensile tests can be well explained by the DIC technique, which gives an important idea for practical ductility analyses of Ti/Al clad sheets. This result is an outstanding one, which has been unreported in previous studies on Ti/Al clad sheets. Since the stress-relief annealing is actually involved in commercial processes of 5052 Al alloy, any additional processing equipments are not needed. Since the present twin-roll-cast Ti/Al clad sheets have excellent tensile properties as well as economic advantages, they provide new applications to lightweight-alloy clad sheets requiring excellent formability and corrosion resistance as well as alloy cost saving. In order to further modify microstructures and properties of twin-roll-cast clad sheets, intensive studies on selecting new alloy combinations, on achieving optimized twin-roll-casting conditions, and on clarifying mechanisms involved in enhanced properties should be continued in the future.

## Methods

### Fabrication of twin-roll-cast Ti/Al clad sheets

A pure Ti and a 5052 Al alloy (composition; Al-0.25Si-0.40Fe-0.10Cu-0.10Mn-(2.2~2.8)Mg-0.10Zn-(0.15~0.35)Cr (wt.%)) were used for the fabrication of twin-roll-cast Ti/Al clad sheets. A vertical twin-roll caster consisted of twin rolls (diameter; 400 mm, length; 200 mm, torque; 15 hp), a crucible, a tundish (diameter; 72 mm, depth; 60 mm), and a pure-Ti-sheet uncoiler. The 5052 Al alloy was molten at 680 °C in a crucible by an induction-melting method. The Al melt was transferred to a tundish, and was cast with a Ti sheet (thickness; 0.5 mm, width; 68 mm) to fabricated 3.0-mm-thick twin-roll-cast Ti/Al clad sheets by using the vertical twin-roll caster.

During fabrication processes of Ti/Al clad sheets, the Al melt was contacted directly with a Ti sheet and a roll whose thermal conductivity was higher than a pure Ti. Large-diameter rolls installed with an interior water-circulating system were used because the solidification of Al melt might be insufficient. The Al melt temperature was maintained as low as possible by using a cooling slope which is quite effective in the low-temperature Al casting^32^, and the time of contacting the Al melt with the Ti sheet was increased by reducing the roll gap and rolling speed to sufficiently solidify the Al melt. This low-temperature Al melting was also useful for preventing the formation of Ti-Al intermetallic compounds such as TiAl_3_, TiAl, and Ti_3_Al^25^. The roll gap and speed were set at 3.0 mm and 30 mm/sec (4.4 rpm), respectively. When the roll speed was too slow, the Al melt could be rapidly solidified before it passed through the rolls. In an opposite case, the Ti sheet might be overheated or locally molten.

The twin-roll-cast clad sheets were annealed at 350~600 °C for 10~60 min in consideration of O-temper treatment of commercial 5052 Al alloy (annealing at 345~415 °C)^25^ in order to relieve stresses formed inside the cast Al substrate and to improve the bonding strength of Ti/Al interface. The objective of 600 °C-annealing was to examine effects of formation of Ti-Al intermetallic compounds on tensile properties. For convenience, the Ti/Al clad sheet specimens annealed at 350 °C, 400 °C, 500 °C, and 600 °C for 10 min, 30 min, and 60 min are referred to as ‘TA31′, ‘TA33′, ‘TA36′, ‘TA41′, ‘TA43′, ‘TA46′, ‘TA51′, ‘TA53′, ‘TA56′, ‘TA61′, ‘TA63′, and ‘TA66′, respectively, for convenience.

### Microstructural characterization and tensile test

These Ti/Al clad specimens were mechanically polished with a diamond paste (size; 1 μm), and microstructures of longitudinal-short-transverse (L-S) plane were observed by using an optical microscope and a scanning electron microscope (SEM, JSM-6330F, JEOL, Japan). Phases present in the clad specimens were identified by X-ray diffraction (XRD, Cu K_α_ radiation, scan rate; 2 deg min^−1^, scan step size; 0.02 deg). Interfacial Ti-Al intermetallic compound phases were examined by using energy dispersive spectroscopy (EDS). Sheet-type tensile bars (gage length; 20.0 mm, width; 5.0 mm, thickness; 3.0 mm) prepared along the longitudinal direction were tested at a strain rate of 10^−3^ s^−1^ at room temperature by using a 100-kN-capacity universal testing machine (Instron 1361, Instron Corp., Canton, MA, USA).

### Digital image correlation

In order to measure the deformation amount including localized tensile strain distribution, a digital image correlation (DIC) technique was used^33^. The specimen surface was photographed by using two cameras (model: Phantom V7.3, Komi, Korea), and a vision strain gauge system (model; ARAMIS 5 M, GOM optical measuring techniques, Germany) was used for detecting 3-dimensional coordinates of a deforming specimen surface. White- and black-color lacquers (model: Aqua, Motip Dupli, Germany) were sprayed on the L-S plane of the tensile specimen to obtain random black-and-white speckled patterns, which were then used for the strain distribution analysis. The surface was recognized by digital camera images, while coordinates were allocated by pixel images, and deformed surface images were sequentially recorded during the tensile deformation. Nominal and local strains at the center position of specimen gage section were measured along the base line (center line of gage section).
